# Intermittent breaking of isolation may ameliorate decrease in physical activity caused by isolation

**DOI:** 10.1371/journal.pone.0314262

**Published:** 2024-11-27

**Authors:** Aritoshi Uchida, Kazuharu Nakagawa, Kanako Yoshimi, Yuki Nagasawa, Kohei Yamaguchi, Naofumi Uesaka, Haruka Tohara

**Affiliations:** 1 Department of Dysphagia Rehabilitation, Division of Gerontology and Gerodontology, Graduate School of Medical and Dental Sciences, Institute of Science Tokyo, Tokyo, Japan; 2 Department of Neurophysiology, Graduate School of Medical and Dental Sciences, Institute of Science Tokyo, Tokyo, Japan; University of Tartu, ESTONIA

## Abstract

Social isolation affects physical functioning owing to psychological stress. We constructed a rat model to clarify the unexplored effects of social isolation and to determine whether environmental changes as an intervention against social isolation can reduce the stress-inducing effects of social isolation on physiological factors. Eight-week-old male rats were divided into three groups: group-housed, isolated, and intervention. Group-housed rats were kept 2 animals per cage. Isolated rats were kept 1 rat per cage. The intervention group alternated between the isolation and group-housed conditions. All rats were euthanized after 21 days. Their plasma, masseter muscles, and lower limb muscles were collected. Body weight, food intake, locomotor activity, muscle weight, and plasma corticosterone, ghrelin, and myostatin levels were measured. The results indicated that there were no significant differences between the group-housed and intervention groups for all outcomes. However, weight gain, food intake, and plasma corticosterone levels were higher in the isolated group than in the group-housed group. Plasma myostatin levels were higher in the isolated group than in the intervention group. Plasma ghrelin concentrations were lower in the isolated group than in the group-housed or intervention groups. In the isolated group, locomotor activity decreased compared to that in the intervention group. The lower limb muscle weight ratio also decreased in the isolated group compared to that in the group-housed and intervention groups. In conclusion, isolation decreased physical activity and affected body weight, food intake, and muscle weight; these changes were associated with corticosterone as a stress marker, ghrelin as an appetite-related factor, and myostatin, which is a growth inhibitor of skeletal muscles. Moreover, these changes were suppressed when the isolation time was reduced in the intervention group. The present study suggests that intermittent breaking of isolation may reduce the physical effects of isolation.

## Introduction

Social isolation in humans leads to decreased well-being, depression, increased mortality, and, especially in the elderly, cardiovascular disease, cerebrovascular disease, and cognitive decline [1–5]. In addition, changes in eating habits, decreased activity, and reduced walking speed and ADLs (Activities of Daily Living) may be associated with physical frailty [6–9].

Social isolation is also considered to be a factor in social frailty, and the presence of social frailty has been reported to affect diet quality, food intake, and nutritional status, especially in elderly men, suggesting that it may contribute to the risk of low nutrition [10]. Recently, some studies have found that increased physical frailty is due to changes in eating habits and decreased physical activity levels, which were caused by increased social isolation during the coronavirus disease 2019 pandemic; therefore, support is needed to prevent undernutrition, including the elimination of social isolation [11, 12]. As people age, death and disability increase among their social circles, and the size of their social networks shrinks. For this reason, social isolation is often unavoidable [13, 14]. Therefore, methods that reduce the effects of social isolation on those living in isolated environments are needed.

In several studies, the effects of social isolation were found to be intricately related to a variety of confounding factors; including living environment; geographic factors related to where the person resides; social resource factors, such as relatives and friends; and economic factors [14]. Because social isolation is complicated by confounding factors, clinical research with human participants is limited. On the other hand, animal experiments can reproduce the isolated state model without considering multiple confounding factors. Previous animal studies have demonstrated that chronic psychological stress in isolation affects body weight, food intake, and physical activity. Depending on age and sex, varying effects have been observed, which have been suggested to be due to peripheral and central ghrelin and adaptive capacity [15, 16]. Previous studies have not sufficiently examined the effects of social isolation on the body and the effectiveness of environmental changes for rats of any age. Studies with older rats are needed; however, as in similar studies [17, 18], we chose to study young rats first as a preliminary study.

The purpose of this study was to clarify the unexplored effects of social isolation and to determine whether environmental changes as an intervention against social isolation can reduce the stress-inducing effects of social isolation on physiological factors.

## Methods

### Treatment of rat

#### Ethics statement

This study was conducted in accordance with ARRIVE guidelines and was approved by the TMDU Animal Ethics Committee.

approval ID: A2023-006A

#### Treatment

Twenty-four 6-week-old male Sprague-Dawley rats were obtained from Sankyo Lab Service in Japan. All rats were acclimatized (two rats/cage) for 14 days (2 weeks) before the start of the experiment. Next, a nanotag (KISSEI COMTEC Co., Nagano, Japan) for measuring spontaneous locomotion was implanted into the abdominal cavity of each rat on the day the experiment began. The nanotag is an implantable locomotion measurement device with a built-in 3-axis acceleration and temperature sensors that measures locomotion by sensing vibrations during exercise and body temperature by sensing the temperature inside the animal’s body [19]. The rats were placed in a holding apparatus, isoflurane (Fujifilm Wako Pure Chemicals Corporation, Japan) was inhaled, and a triad of anesthesia (5.0 mL/kg) was administered subcutaneously. The triad of anesthetics consisted of medetomidine hydrochloride (Orion Corporation, Japan), midazolam (Sandoz K.K., Japan), and butorphanol tartrate (Meiji Seika Pharma Corporation, Japan). After shaving the abdomen, an approximately 1 cm incision was made, and the nanotag was implanted and sutured with a silk thread (3 to 5 stitches). After suturing, the rats were awakened by subcutaneous administration of the antibacterial drug ampicillin sodium (100 mg/kg) to the abdomen and the anesthetic antagonist medetomidine (5.0 mL/kg).

After the experimental period is over, Rats were sacrificed by cardiac puncture using a 10 ml sterile syringe and winged needle under deep anesthesia with isoflurane. Anesthesia was administered during surgery and euthanasia, and sufficient care was taken to minimize animal suffering.

### Experimental animals and social groupings

After nanotag implantation, the animals were kept for 3 weeks in three groups [20]. Rats in the “group-housed (Control)” group (n = 8) were kept in 2 animals/cage. Rats in the “isolated (Iso)” group (n = 8) were kept in 1 rat/cage. The sides of the cages were covered with gray cardboard to create isolated situations so that rats in neighboring cages could not see each other. In the “Intervention (Int) “group (n = 8), the group-housed and isolated conditions were alternated every other day. All rats were kept in a clean room at 23 ± 2°C, 55% ± 10% relative humidity, with a 12-h light/dark cycle, and the lights were on from 8:00 to 20:00. The rats were provided food and water ad libitum throughout the study.

### Data collection

#### Weight gain rate and food intake

The body weight and food intake of the three groups were measured every other day from the beginning of the experiment.

The weight gain rate was calculated based on the body weight on the first day of the experiment. Food intake was calculated by weighing the amount of feed remaining in the cages. For the group-housed rats, the total amount of food consumed was averaged between the rats [15].

#### Locomotor activity

The nanotag, a spontaneous locomotor measurement device implanted in the abdominal cavity of each rat, was retrieved from each rat during autopsy on the last day of the experiment. Locomotor activity was measured during the light and dark phases [19].

#### Muscle to body weight ratio

On day 21 of the experiment, after the rats were euthanized, the masseter and lower limb muscles (gastrocnemius and soleus muscles combined) were removed, washed in saline, and weighed after excess water was removed with filter paper. At the end of the experiment, the percentage of muscle weight to body weight was calculated [21].

#### Determination of plasma proteins by enzyme-linked immunosorbent assay (ELISA)

Rats were fasted for 8 h prior to euthanasia and were subjected to restraint stress [22] with a strait jacket for 30 min immediately prior to autopsy. Isoflurane inhalation anesthesia was used, and whole blood was collected by direct cardiac puncture under deep anesthesia. The collected blood was immediately centrifuged with heparin blood coagulant, and plasma was extracted and stored at -80°C. Plasma levels of corticosterone, myostatin (growth/differentiation factor-8: GDF-8), and ghrelin were quantified by ELISA using the Parameter Corticosterone Assay kit (R&D Systems., Minneapolis, MN, USA), GDF-8/Myostatin Quantikine ELISA kit (R&D Systems), and YK350 Active Ghrelin ELISA kit (Yanaihara Laboratories., Shizuoka, Japan), respectively. ELISA was performed according to the manufacturers’ instructions.

### Data analysis

The results of weight gain rate, food intake, physical activity, muscle weight to body weight ratio, and plasma protein concentration were statistically analyzed for differences among the three groups. All items were compared among the three groups using Tukey’s test (significance level, *p <* 0.05) after confirming equal variance. Statistical analyses were performed using SPSS software version 28.0.1.0 (IBM Corp., Armonk, NY, USA).

## Results

### Weight gain rate (Fig 1A)

**Fig 1:**
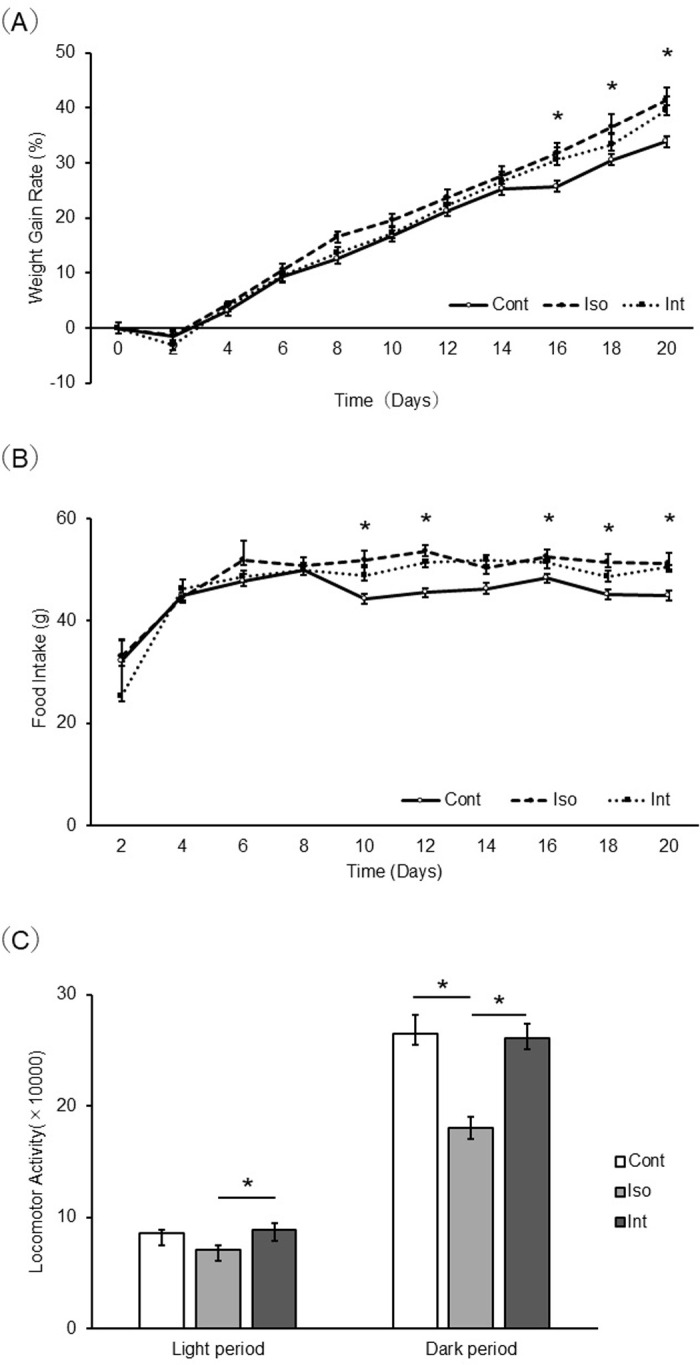
Comparison of weight gain rate, food intake, locomotor activity between the Control, Iso, and Int groups. (a) Weight gain rate (b) Food intake (c) Locomotor activity (n = 7–8). *p < 0.05 (Tukey’s test). Control group, group-housed; iso group, isolated; Int, intervention.

There were no significant differences between the Int and Control groups with respect to the rate of weight gain.

In contrast, the Iso group had a significantly higher weight gain from the start of the experiment to day 20, just before the end of the experiment, compared to the Control group on days 16, 18, and 20 (*p* = 0.007, 0.012, 0.01, respectively).

### Food intake (Fig 1B)

Food intake was not significantly different between the Int and Control groups. On the other hand, food intake in the Iso group increased significantly after day 10 compared to that in the Control group.

### Locomotor activity (Fig 1C)

Locomotor activity determined by nanotag analysis was found to be significantly reduced in the Iso group compared to that in the Int group during the light period (*p* = 0.023). In the dark phase, the active phase of the rats in the Iso group showed a significant decrease compared to that in both the Control and Int groups (*p* < 0.001).

### Muscle to body weight ratio (Fig 2)

**Fig 2:**
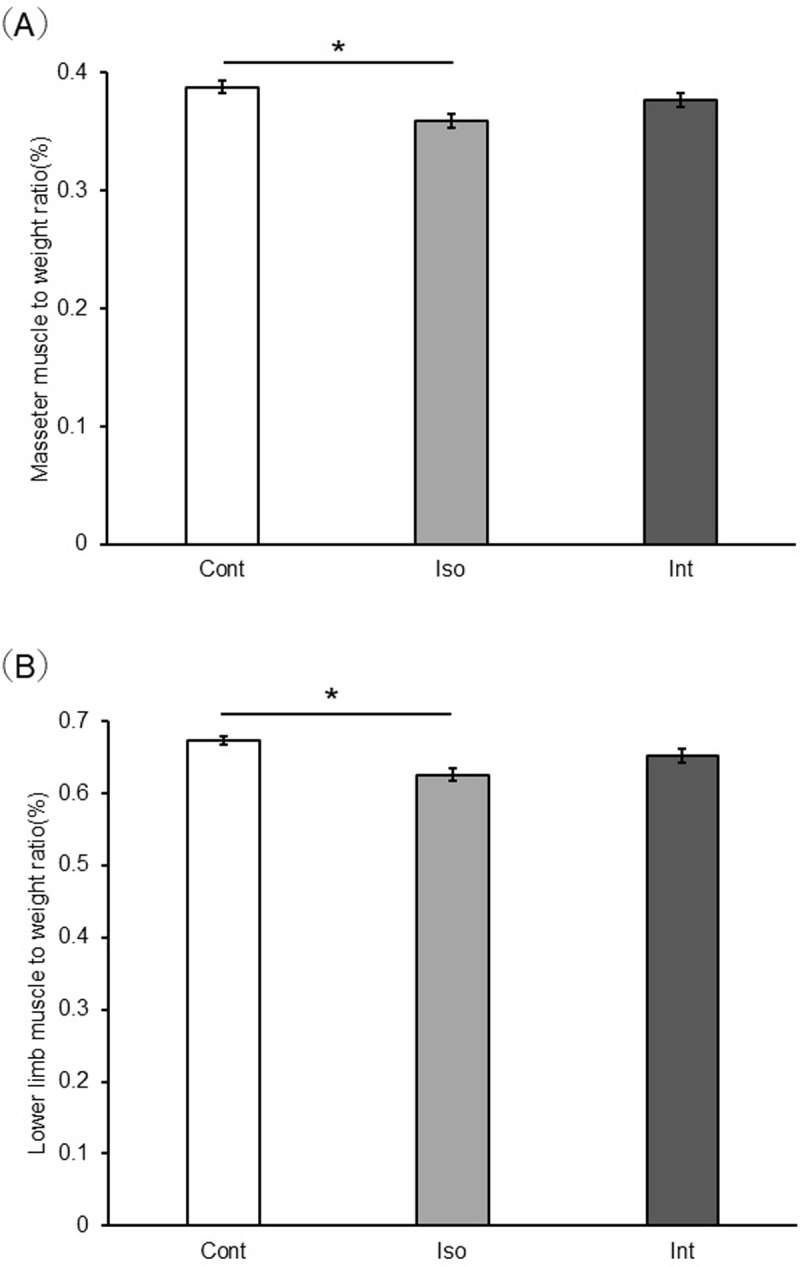
Comparison of muscle weight to body weight ratio between the Control, Iso, and Int groups. (a) Masseter muscle (b) Lower limb muscle (n = 8). *p < 0.05 (Tukey’s test). Control group, group-housed; iso group, isolated; Int, intervention.

The masseter muscle-to-body weight ratio was not significantly different between the Int and Control groups. On the other hand, the ratio in the Iso group was significantly lower than the Control group (p = 0.002). Similarly, the lower extremity muscle/body weight ratio in the Iso group was significantly lower than the Control group (*p* = 0.004).

### Plasma protein concentration (Fig 3)

**Fig 3:**
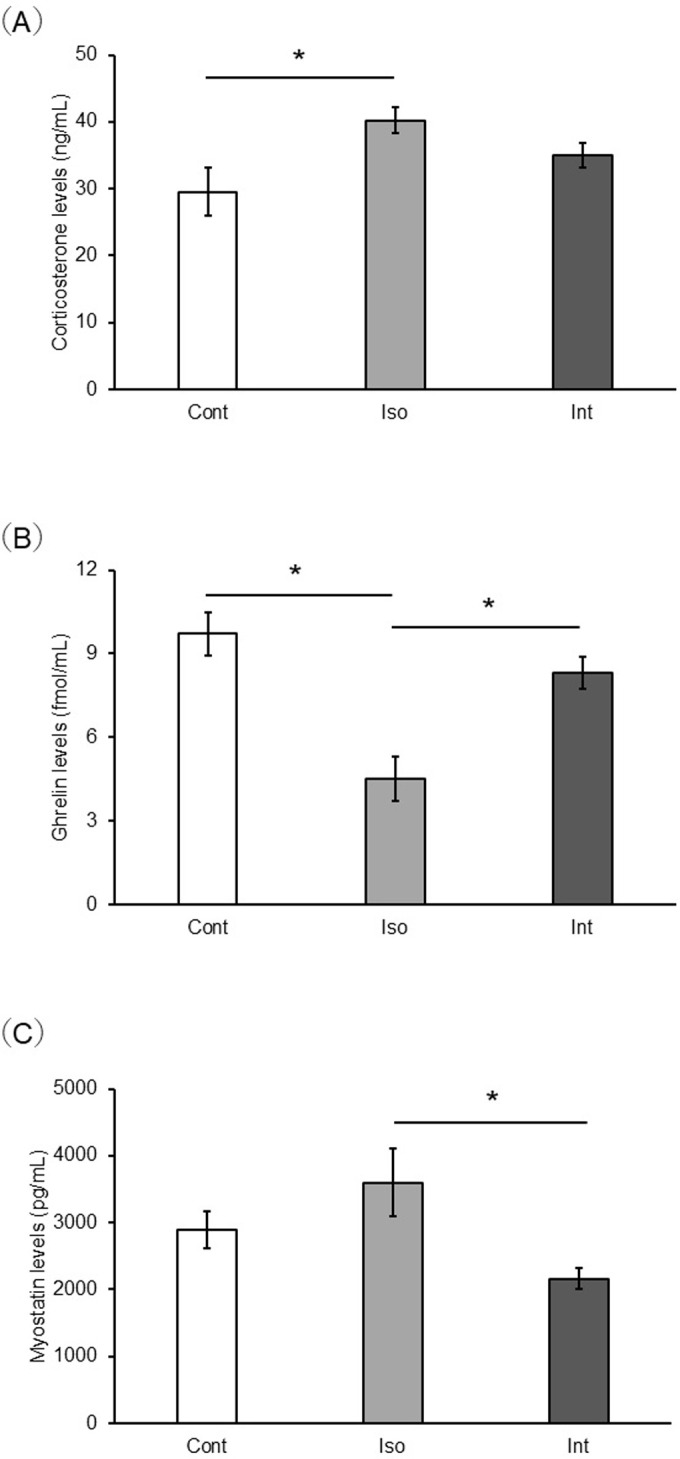
Comparison of plasma corticosterone, ghrelin, and myostatin concentrations between the Control, Iso, and Int groups. (a) corticosterone (b) ghrelin (c) myostatin (n = 4). *p < 0.05 (Tukey’s test). Control group, group-housed; iso group, isolated; Int, intervention.

Plasma corticosterone levels were not significantly different between the Int and Control groups. However, there was significant difference between the Iso and Control groups with respect to the plasma concentration (*p* = 0.033). Plasma acylated ghrelin levels were significantly lower in the Iso group than in the Control and Int groups (*p* = 0.001 and 0.009, respectively). Plasma myostatin levels were not significantly different between the Int and Control groups. However, plasma myostatin levels were significantly higher in the Iso group than in the Int group (*p* = 0.03).

## Discussion

The present study suggests that intermittently alternating rats between isolation and group housing may significantly reduce the decrease in locomotor activity and improve the masseter muscle and lower limb muscle to body weight ratios observed in isolated rats. Several studies have shown that isolation affects body weight, food intake, ghrelin and corticosterone levels, and physical activity [15, 21, 23, 24].

Weight gain and food intake increased significantly in the Iso group compared to those in the Control group. This was consistent with previous reports showing that the rate of weight gain and food intake increased in association with isolation status in young rats [15]. Although no significant differences were observed between the Iso and Int groups, the intervention tended to suppress the rates of weight gain and food intake. In this study, as in previous studies on isolation, all groups were kept in the same cage size, with two animals per cage for the control group and one animal per cage for the Iso group [25–27]. Previous studies have reported that larger cages and increased cage density are associated with decreased body weight and some organ weights [28]. On the other hand, some reports indicate that deviations or exceptions from the cage density and sanitation frequency standards specified in the guidelines do not adversely affect animal health or welfare [29, 30]. Thus, the optimal number of rats per group has not yet been established. The results of this study may be influenced by cage density.

Isolation can also affect several plasma hormone levels. Ghrelin is a peptide consisting of 28 amino acid residues released from the stomach and intestinal tract to promote appetite [31]. There are two forms of ghrelin: acylated ghrelin (AG) and unacylated ghrelin (UAG), with ghrelin acylated by octanoic acid modification being the active form [32]. Acylated ghrelin is produced primarily by cells in the oxygenated glands of the human and rat stomachs [33], and it increases appetite and food intake by activating the hypothalamic arch nucleus [34]. Plasma ghrelin levels increase during fasting and decrease after eating [35, 36]. Body weight gain due to overfeeding in rats decreases plasma ghrelin levels [37]. In obese individuals, plasma ghrelin levels are decreased and negatively correlated with body fat percentage and body mass index [38–40]. In this study, plasma ghrelin levels were significantly lower in the Iso group than that in the Control group. Isolation may have affected plasma ghrelin levels as well as the rate of weight gain and increased food intake. In addition, plasma ghrelin levels were significantly lower in the Iso group than in the Int group. The changes caused by isolation may be mitigated by intermittent deisolation.

Corticosterone is a glucocorticoid in rodents that is released in response to circadian rhythms and stress responses [23] and has been reported to influence feeding behavior, especially increased food intake and weight gain [41–43]. In this study, plasma corticosterone concentrations were significantly higher in the Iso group than in the Control group. Although there was no significant difference between the Iso and Int groups, the intervention tended to reduce the increase in the plasma corticosterone concentration. Therefore, the increased food intake and rate of weight gain in the Iso group observed in this study may be related to changes in plasma ghrelin and corticosterone concentrations.

Myostatin, or GDF-8, is a protein produced by skeletal muscles and acts as a hormone [44]. This hormone is a negative regulator of skeletal muscle growth, and skeletal muscle mass decreases as plasma myostatin levels increase [45]. Myostatin also has an inhibitory effect on skeletal muscle growth and promotes fat accumulation [46, 47]. Plasma myostatin levels were significantly higher in the Iso group than in the Control and Int groups. The masseter muscle to body weight ratio was significantly lower in the Iso group than that in the Control group, and the lower extremity muscle to body weight ratio was significantly lower in the Iso group than that in both the Control and Int groups. This suggests that the isolation conditions may have affected the factors that inhibit skeletal muscle proliferation, resulting in a loss of skeletal muscle relative to body weight. This also suggests that group living, even if intermittent, may inhibit the effects of isolation on skeletal muscles.

During the dark period, locomotor activity in the Iso group was significantly reduced compared to that in the Control and Int groups. During the light period, locomotor activity in the Iso group was significantly reduced compared to that in the Int group. Previous studies have suggested that social isolation causes reduced physical activity [19], and increased physical activity has been associated with improvements in various diseases, including coronary artery disease, hypertension, dementia, stroke, diabetes, osteoporosis, and depression [48–50]. Therefore, maintaining physical activity levels is important.

In the Int group, rats were intermittently released from isolation reducing the isolation time by half. Halving the time in complete isolation significantly suppressed the changes in locomotor activity, skeletal muscle mass, food intake, and body weight gain that occurred during isolation, accompanied by dynamic changes in the appetite-related factor ghrelin and the skeletal muscle growth suppressor myostatin. Social isolation among older adults is becoming increasingly severe every year [51]. In clinical practice, we often encounter young people with disabilities and older people living alone who have reduced ADLs and activity levels. Previous studies examining social isolation during the coronavirus 2019 pandemic have suggested a negative correlation between the length of isolation time and memory and cognitive function [52]; therefore, reducing isolation time may be of high clinical significance. This study suggests that an intermittent reduction in isolation time by half, even if not long-term group living, may reduce the physical effects of isolation.

This study is the first to report the effectiveness of an intervention method that intermittently reduces isolation time. It is not yet clear to what extent interventions for individuals in social isolation are effective in reducing the effects of social isolation and maintaining quality of life when applied to humans; however, the results may help when considering measures to prevent social isolation.

## Conclusion

The present study suggests that rats in isolation experienced an increased rate of weight gain and food intake and a decrease in locomotor activity and skeletal muscle percentage of body weight, and that these changes were related to the stress marker corticosterone, appetite stimulator ghrelin, and skeletal muscle growth suppressor myostatin. We found that intervention methods that intermittently return isolated rats to group housing may reduce the physical effects of isolation.
